# Study on the Influence of Selected Fabrics and Stitching on the Strength of Upholstery Covers

**DOI:** 10.3390/ma15113854

**Published:** 2022-05-28

**Authors:** Julia Lange, Agata Ciura, Adam Majewski, Marlena Wojnowska

**Affiliations:** Department of Furniture Design, Faculty of Forestry and Wood Technology, Poznan University of Life Sciences, 60-637 Poznan, Poland; ciura.agata@wp.pl (A.C.); adam.majewski@up.poznan.pl (A.M.); marlena.wojnowska@up.poznan.pl (M.W.)

**Keywords:** upholstery, sewing, furniture, seam, strength, design

## Abstract

So far, no coherent and comprehensive method has been elaborated allowing investigation of tensile strength of upholstery seams dedicated to upholstered furniture. Producers of this type of furniture are interested in the assessment of the quality of upholstery material joints, which seems to be particularly important for ensuring the appropriate quality of products. Therefore, the objective of this research was to investigate the influence of the type of material used and the direction of the fabric cut on the strength of upholstery covers. Static tensile testing of selected upholstery fabric samples was performed, and an attempt was made to identify the most optimal fabric–seam joints. It was stated as a conclusion that the fabric tensile strength was the highest for Secret 10 fabric. In addition, the strength of upholstery covers is not influenced by the direction of the fabric die cut. For each fabric, a different configuration is preferable, as shown by results (Power 13: A-B, Secret 10: B-B, Soft 10: A-A). The method, implemented for upholstered furniture, allows for an objective assessment of the strength of upholstery covers and the selection of the most advantageous fabric–seam combination for future furniture designs.

## 1. Introduction

Furniture is a basic object for everyday use and is both practical and functional in character, providing also decorative value to residential interiors. Upholstered furniture, apart from the main relaxation aspect, is designed to make the interior more attractive. The complexity of upholstered furniture construction allows freedom in designing forms and shapes, and thus creation of unique and unrepeatable objects. The aesthetic appearance of furniture is mainly affected by the quality of workmanship. However, during the production process there is a risk of defects appearing, which significantly reduces the aesthetic value of the furniture and decreases the product’s impact on the customer. Moreover, upholstered products should be characterized by durability in both construction and cover elements, which are in direct contact with the human body, maintaining comfort and safety of use. Such technologically and structurally complicated products as upholstered furniture are subjected to detailed quality control, especially in terms of fabrics. When considering only the textile aspect, it must be stated that textile production is one of the oldest worldwide industries, with a production volume exceeding 88.5 million tons per year. It is a matter of great importance, especially for developing countries whose share reached 58.6% of the global textile market [1]. Fabrics, as a fundamental material for, e.g., clothing, provide an extended research field, including: types, structure, mechanical and acoustic properties, comfort, and the manufacturing production process, as well as environmental impact. Thus, in the literature of the subject, comprehensive research on textile physical testing methods and advanced characteristics can be found [2,3]. In general, the quality of the fabric is, on the one hand, described based on such parameters as tensile strength, elongation percentage, rupture, texture and breathability. On the other hand, it is also affected by the manufacturing method and environmental conditions. It is also stated that fabric strength results from the type of raw materials, yarn characteristic, spinning technique, fabric geometry, yarn crimp during processing, and weaving conditions (including temperature, humidity, and yarn tensions during weaving) [4]. Researchers examined textiles, taking into consideration numerous intersecting aspects. Some of them tested textiles experimentally [5,6,7,8,9]. Ramasamy et al. [10] investigated the effects of the composition of tencel fiber in a cotton/tencel blend based on yarn-on-yarn strength, elongation, yarn diameter, packing fraction, hairiness, and frictional properties. They concluded that the addition of tencel fibers increases the strength of the blend yarn to a significant level, with higher tencel composition. Elongation of the blend yarn increases slightly with the increase in tencel composition. Others assessed pilling—one of the most common surface defects in polyester–cotton blended fleece fabrics [11]. Authors concluded that singeing and heat setting are the most effective methods for reducing pilling in polyester–cotton-blended, three-thread fleece cloth. Inspiring findings were presented by Witczak et al. [12]. Authors tested, i.a., the acoustic properties (sound absorption coefficient) of flat, relief and jacquard textiles. As a conclusion, it was mentioned that the more loosely woven the fibrous structure, the better acoustic absorption occurs. A similar effect was observed for yarn creating the fabric. The lower the yarn linear density and thinner the fibers, the better the sound absorption. It must be also emphasized that there is a significant connection between the environment and the apparel industry; hence, textile production can cause significant damage to the environment [13]. As was discovered by Wang et al. [14], the textile companies of developing countries should responsibly choose the appropriate technology that results in emission reduction, reduces their cost of emission reduction and reduces the product differences with the developed country firms. Ranasinghe and Jayasooriya [15] presented a comprehensive literature review on textile ecolabelling by accessing the “Ecolabel Index” database. A total of 107 ecolabels existing in the textile industry were studied. Authors exhibited the importance of focusing on regional or ‘gate-to-gate’ ecolabelling frameworks, due to the outspread nature of the product manufacturing life cycle that covers a range of geographical regions. Others performed a life-cycle assessment of textiles made of cotton, polyester, nylon, acryl, or elastane [16]. In terms of waste, a promising outcome was also revealed [17]. The authors concluded that 100% of textile waste can be used for making yarn, in order to reduce environmental impact and to achieve a product at a low cost. These yarns can be used for manufacturing denim, towels and chino cloth for trousers. The presented brief literature review shows that many researchers analyzed textiles in the context of a finished product. Apart from the fabric itself, the aspect of seams is also important. Some researchers tested seam stiffness and strength efficiency along with the draping behavior and seam puckering. Samples were made from three types of commercially available fabrics of medium weight construction, varying in the blend composition of polyester and cotton components [18]. Bhavesh et al. [19] investigated the impact of weft knitted fabric structures, sewing thread types and stitch types on seam strength and efficiency of the superimposed type of seam for cotton apparel. They proved that higher strength of thread results in higher seam strength and better functional performance of seam. Seam strength increases with the increase in sewing thread linear density. The effect of stitch length was also studied [20]. The authors concluded that sewability (indicated by penetration force) of 100% cotton, single-jersey fabric is inversely proportional to its stitch length. Moreover, fabrics with coarser yarn counts and shorter stitch lengths have better functional performance characteristics. Yesmin et al. (2014) [21] reported, e.g., that the bursting strength of tested fabric gradually decreases with the increase in stitch length. Therefore, maximum bursting strength is achieved in small stitch lengths.

The covering material, a structure built from connected pieces of fabric, is a key element in the aesthetics and appearance of upholstered furniture. The quality of the stitching and the way the fabric fits on the cover are crucial for the overall external appearance of the product. Testing the strength of upholstery covers requires consideration of many aspects that build the fabric–joint system. The strength of upholstery covers is influenced by several factors, such as: the type of fabrics used, cover type of construction and direction of cutting, the type of threads used, the type of stitching and seam density. Unfortunately, it was observed that the cover, as the outer layer of upholstery systems, is frequently neglected during testing. Quality checking is often limited to applying force simply by hand and through seam observation. This method is insufficient to more extensively counteract potential defects in cover stitching. In everyday use, the reliability of the cover’s connections is just as crucial as the internal construction of the entire piece of furniture since the cover is in direct contact with the user. Thus, it is exposed to the greatest damage. Furniture producers are looking for test methods that allow them to obtain the best possible configuration in terms of strength, which also would minimize the occurrence of potential customer complaints.

In addition, the need for research development concerning the strength of upholstery covers is a consequence of the scarce number of publications covering fabrics in general (considered as the outer layer of upholstery systems). From the literature review, it is evident that available scientific works concern mainly the analysis of upholstery systems: springs [22,23] and foams [24,25,26,27,28,29,30,31,32,33,34,35]. Publications dealing with fabrics used in the production of upholstered furniture most often concern fire safety of housing and furniture flammability [36,37,38,39,40]. The most related publication to this research deals with a different characteristic—abrasion [41]. However, this publication does not consider upholstery fabrics in terms of seam strength.

Determining the influence of selected factors on the strength of furniture covers will allow the proper selection of materials at the early stage of the upholstered furniture design process and the elimination of manufacturing defects of finished products. The knowledge gained may be particularly useful in increasing the quality of upholstered furniture, thus reducing the number of complaints in the field of sewing work and increasing the durability of products and safety of use. Therefore, the aim of the study was to investigate the effect of the direction of the fabric die cut and the type of material used on the seam strength and failure form.

## 2. Materials and Methods

Samples were prepared in such a way to consider how different components affect the strength of fabric–seam joints. The fabric samples were paired in different configurations. A fabric is a flat textile product formed from two arrangements of threads oriented perpendicularly to each other and interwoven according to a specific order [42]. When a fabric is made, an arrangement network of threads is formed, arranged alternately in an orderly manner. The longitudinal threads are the warp, the transverse threads are the weft, and the way they are connected is the weave [43]. The fabric, as in the case of wood, has a different structure depending on the direction of cutting. Therefore, the specimens were related to the direction of cutting, as follows: A-A, B-B and A-B (where A—along the warp direction; B—across the warp direction, along the weft).

The following fabrics were chosen for the study (Table 1): Power 13, Secret 10, Soft 10, as types popularly used in the furniture industry. The most common type of thread used in upholstery furniture production is type 20 thickness thread. In industrial and commercial practice, the gravimetric method is used, which means that the thickness is indicated by a number (e.g.: 20; 40). The number determines the ratio of the thread weight to its length [39]. The higher the designation, the thinner the thread, as follows: coarse threads—10–24 Nm; medium threads—30–50 Nm; fine threads—60 or more Nm.

When selecting threads for individual sewing operations, attention should be paid to their thickness. For sewing upholstery covers, which are used directly and are exposed to heavy loads (e.g., backrest, seat), thicker threads are used, whereas in decorative seams (e.g., for sewing decorative cushions) threads of smaller thickness are chosen—the seam looks more aesthetic and does not require as much strength as in the case of seat or backrest covers. An important parameter of yarn structure is also its twist degree, i.e., the number of twists per unit length. As the twist of the yarn increases, its strength increases, and thus so does its resistance to tearing, which is of vital importance in the production of upholstery covers. Due to the utilitarian nature of products, where sewing threads are used, they must be characterized by high strength, dimensional stability during use and resistance to discoloration under the influence of various factors.

Stitch type and stitch length—straight lockstitch with a stitch length of 3 mm—were the constant parameters in the conducted research. The samples were sewn together on a Juki sewing machine (JUKI Corporation, Tokyo, Japan) using a 100 needle, which is most often used in the production of upholstered furniture and universal for sewing the fabrics mentioned above.

**Figure 1:**
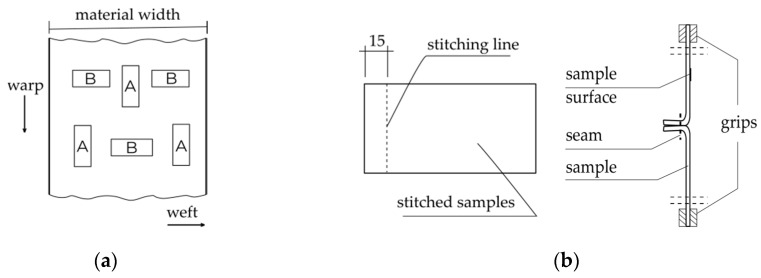
(**a**)—fabric cutting method, (**b**)—fabric strength test method.

Samples of dimensions 50 × 200 mm^2^ were cut according to the requirements of ISO [44] and industry standard [45] (Figure 1a). Due to the lack of standards for seam tensile strength testing of residential furniture fabrics, it was decided to use the upholstery materials test standard for the automotive industry. The fabric specimens were cut along the warp direction (specimen A) and across the warp direction (specimen B), and then right sides were sewn together. The seam line was drawn 10 mm from the edge of both samples. The test was conducted based on the industry standard [45] (Figure 1b). The specimens were subjected to a static tensile test until complete failure. It is a basic experiment of material mechanical properties, which allows the observation and recording of the tensile process from the moment of specimen loading to failure [46].

**Figure 2:**
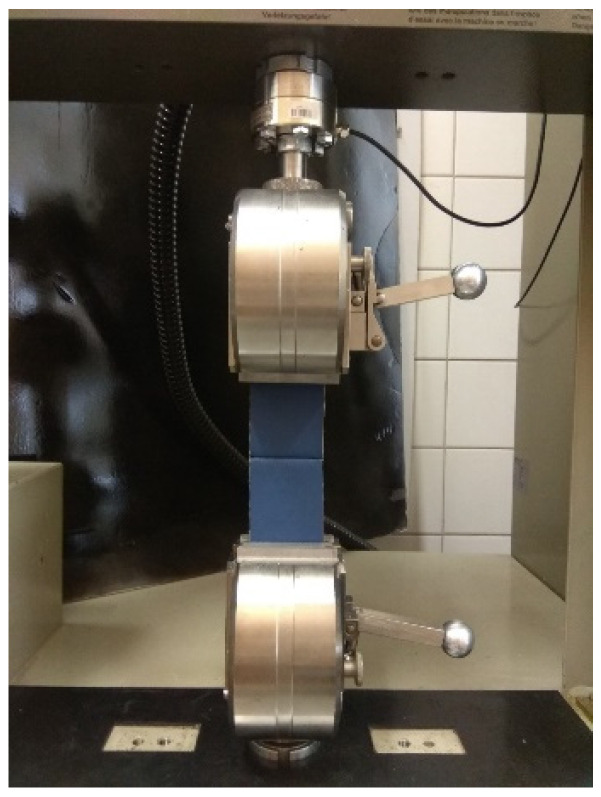
Sample testing station.

The tests were performed using a Zwick 1445 numerically controlled testing machine (Zwick Roell GmbH and Co.KG, Ulm, Germany). Ten specimens from each group (A-A, B-B, A-B) were subjected to tensile tests with identical parameters (Table 2). Each specimen was positioned so that the seam was in the center of the working area, and equal sections of material were placed in the grips on each side to achieve a uniform force distribution (Figure 2). The specimens were stretched until the fabric-sewn system ruptured.

## 3. Results

During the tensile test, the failure force F_(max)_ (N) and displacement ΔF_(max)_ (mm) were measured for each fabric type (Table 3, Table 4 and Table 5). It is worth noting that for sample no.6 of Power 13 fabric, the maximum force reached extraordinary values both in force and displacement. It was a result of having denser sewing and double finishing in one sample side, which was not noticed. This sample test was eventually excluded from further analysis.

**Figure 3:**
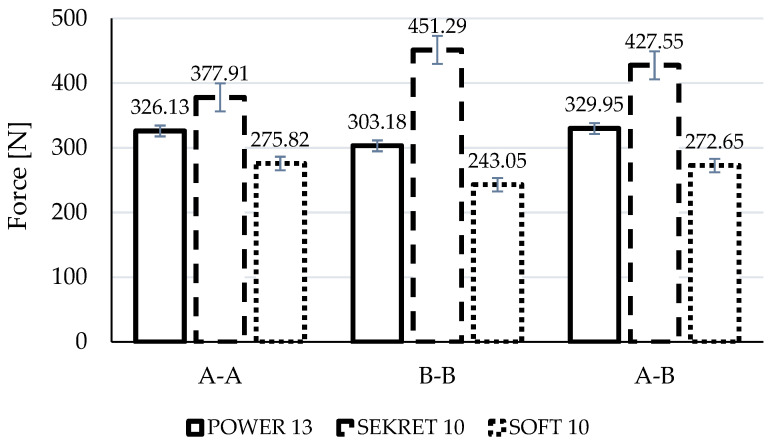
Fabric rigidity in cutting direction: A-A, B-B, A-B.

**Figure 4:**
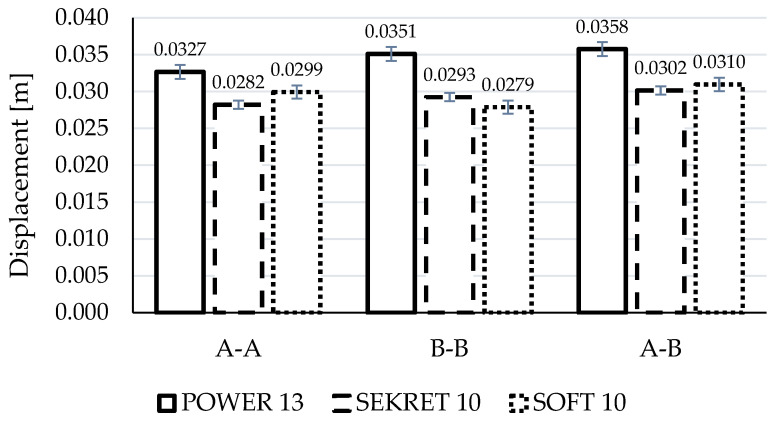
Fabric displacement during test in cutting direction A-A, B-B, A-B.

Figure 3 shows averaged fabric rigidity with the standard deviation of the results taking into consideration the sample’s cutting direction. Figure 4 gathers values of sample displacement during tensile tests.

## 4. Discussion

**Figure 5:**
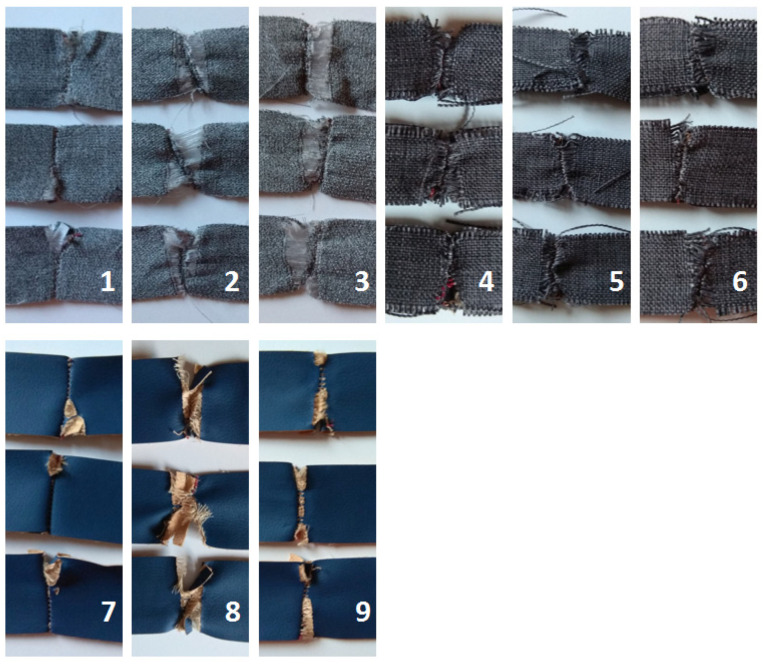
Summary of failure test: (**1**) Power 13 A-A, (**2**) Power 13 B-B, (**3**) Power 13 A-B, (**4**) Secret 10 A-A, (**5**) Secret 10 B-B, (**6**) Secret 10 A-B, (**7**) Soft 10 A-A, (**8**) Soft 10 B-B, (**9**) Soft 10 A-B.

Static tensile tests exposed how various factors affect the strength of upholstery covers. The tests showed the relationship between the different parameters in terms of sewing technique and the type of fabric used. Depending on the fabric type and direction, the failure of the samples within the seam is different (Figure 5).

Based on the analysis of the results, it might be stated that destruction is the least for samples stitched in the warp direction (A-A) for all fabric types (Figure 5 subsections 1, 4, 7), particularly for Power 13. The seam of Power 13 and Soft 10 samples was ripped mainly from the sides, causing relatively little fabric damage. Interesting is the fact that despite the strongest damage to the fabric, the biggest value of damaging force and at the same time the smallest displacement were noted for Secret 10—377.91 N and 0.0282 m—while force values for Power 13 and Soft 10 were less than 16% and 37% and displacement greater than 16% and 6% respectively.

Analyzing the B-B cutting direction results (across the warp direction), one can see a greater destruction of the seam and a significant violation of the fabric along the seam on both sides (Figure 5, subsections 2, 5, 8). In the case of the Power 13, the destruction occurs mainly within the fabric. The Secret 10 and Soft 10 samples had little fabric destruction and mainly the seam was destroyed. Again, Secret 10 exposed the biggest damaging force (451.29 N), being 49% and 86% bigger than Power 13 and Soft 10, respectively. The displacement of Soft 10 was the smallest—0.0279 m—but in comparison to Secret 10, this was only 5% (0.0293 m).

In relation to the A-B direction, it can be stated that Secret 10 fabric showed the biggest value of damaging force (427.55 N) and the smallest displacement (0.0302 m), similarly to the two other cutting configurations (Figure 5, subsections 3, 6, 9). Values for Power 13 and Soft 10 were less, for nearly 30% and 57%, respectively. In contrast to force values, there is a smaller difference in displacement—Power 13 had 18.5% and Soft 10 had 2.6% bigger values than Secret 10. In all die-cut direction cases, the damage was observed rather within the seam.

In general, when considering the samples tested in terms of fabric type, certain correlations can be observed. In the case of Power 13 fabric, which is an elastic material with pile, the most visible is the variation of damage depending on the direction of the sample cutting. These are, in each case, large damages, together with violation and displacement of the material along the seam. Taking the results into consideration, it can be noticed that average values of force F_(max)_ (A-A: 326.13 N, B-B: 303.18 N, A-B: 329.95 N) and displacement _(max)_ (A-A: 0.0327 m, B-B: 0.0351 m, A-B: 0.0358 m) during the tensile test are comparable. The Power 13 fabric has similar strength in all directions, but due to its high stretch ability, the nature of the damage is different depending on the direction of the specimens.

The most durable fabric in all directions with the least amount of destruction is Secret 10. This is a knitted fabric, with an even weave, where the weft and warp are made of the same thread, so the direction of the cut-out has little influence on the seam destruction during the tensile test. It has the greater grammage among tested fabrics (400 g/m^2^). In each case, Secret 10 reached the highest rigidity and behaved in a fairly similar manner, with slight deviations. However, the mean displacement values for all cutting groups are comparable. This demonstrates the relatively equivalent tensile behavior of the material, regardless of the direction of the specimens’ die-cut.

The Soft 10 fabric behaves similarly to the Power 13 fabric, where the seam failure is determined by the direction of the specimen’s die-cut. In both fabric types, the weft and warp are significantly different; hence, the variable form of destruction is closely related to the cutting direction of the samples. Soft 10 fabric showed the lowest strength of all the materials tested. This might result from the fact that it is a skaic fabric, commonly called “eco-leather”. Its unusual structure has a significant impact on its strength. The artificial coating made of polyurethane on a thin backing does not have any weave to reinforce the structure of the material, hence such low F_(max)_ results. However, the damage takes a different form depending on the direction of the tensile force. The displacement, as in the case of the Power 13 fabric, is comparable; the mean values are similar (A-A: 0.0299 m, B-B: 0.0279 m, A-B: 0.0310 m).

## 5. Conclusions

On the basis of the strength analysis of upholstery cover tests, the following conclusions can be formulated:

1. The strength of upholstery covers is influenced by the type of fabric. A knitted material with an even weave, where the weft and warp are made of the same thread is characterized by better tensile rigidity. Furthermore, in this case the seam failure, form does not depend on the die-cut direction. The aforementioned statement was illustrated in the presented study, where Secret 10 fabric was the strongest one, achieving a damaging tensile force of 377.91 N (A-A), 451.29 N (B-B) and 427.55 N (A-B), while displacement was 0.0282 m, 0.0293 m and 0.0302 m, respectively.
2. A sample’s die-cut direction affects the seam failure form, but does not influence the averaged tensile damaging forces and observed displacements. When comparing extreme damaging forces within the same fabric samples, but in different die-cut methods (A-A, B-B, A-B), it might be mentioned that differences are as follows: for Power 13–9%, Secret 10–19% and Soft 10–14%. For displacement, value differences for Power 13, Secret 10 and Soft 10 are 9%, 7%, and 11%, respectively.
3. The seam failure form in different die-cut directions is closely correlated with the fabric construction, where the weft and warp can be made of different thread types. In the case of Power 13 and Soft 10 fabrics, it is evident that, e.g., A-A die-cut direction results in relatively small fabric damage and mainly from sides, while for B-B direction, the seam was greatly damaged.
4. This work can serve as an aid to the industry in the design of soft furnishings and construction of upholstery layouts, contributing to the rational and informed selection of materials to produce upholstery furniture. Based on the analysis of seam damages, it is possible to recognize whether the nature of the damage to the seam-fabric combination is a result of the cover being sewn together incorrectly or is caused by the fabric structure.

## Figures and Tables

**Table 1:** Characteristics of selected upholstery fabric types.

Fabric Name	Secret 10	Power 13	Soft 10
Fabric view	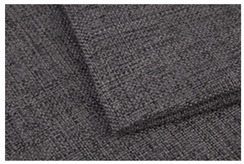	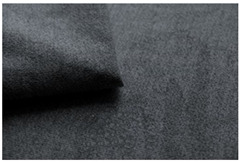	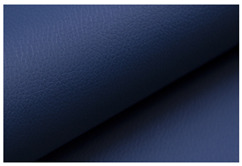
Fabric swatches sewn right side together (as in upholstery covers)	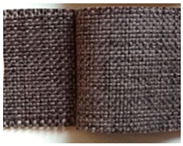	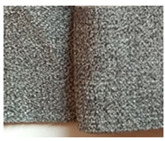	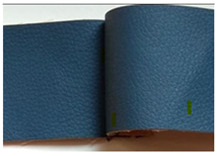
Fabric samples—seam visible on left side of samples	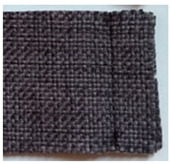	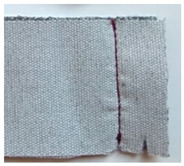	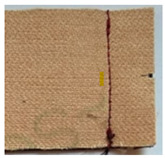
Type	No pile fabric	Pile fabric with textile cover (velvet)	Skaic/artificial leather
weave	canvas	canvas	-
Material	100% polyester	100% polyester	100% polyurethane
Grammage	400 g/m^2^	320 g/m^2^	390 g/m^2^
Thickness [mm]	1.00	1.00	0.95
Abrasion resistance [cycles]	100,000	100,000	100,000
Seam type	straight lockstitch	straight lockstitch	straight lockstitch
Thread type	20	20	20

**Table 2:** Zwick 1445 testing parameters.

Test Type	Tensile
mode	damaging
Max. force loss	100 N
Force stabilizing speed	10 mm/min
System reset force	5 N
Max. deflection	100 mm
Max. working speed	100 mm/min
Max. force	10 kN
Testing speed	10 mm/min

**Table 3:** Power 13 fabric test results.

Sample		1	2	3	4	5	6	7	8	9	10
A-A	F_(max)_ [N]	306.35	338.35	288.35	322.35	286.35	1367.4 *	352.35	370.35	378.35	292.35
	ΔF(_max)_ [m]	0.0613	0.0293	0.0264	0.0268	0.0265	0.1198 *	0.0334	0.0325	0.0320	0.0258
B-B	F_(max)_ [N]	330.35	320.35	258.35	208.68	324.35	296.35	302.35	334.35	324.35	332.35
	ΔF(_max)_ [m]	0.0349	0.0371	0.0323	0.0218	0.0393	0.0327	0.0366	0.0408	0.0385	0.0371
A-B	F_(max)_ [N]	334.35	312.35	326.35	364.35	296.35	334.35	352.35	356.35	324.35	298.35
	ΔF(_max)_ [m]	0.0334	0.0335	0.0364	0.0392	0.0325	0.0348	0.0390	0.0369	0.0331	0.0388

* result was excluded.

**Table 4:** Secret 10 fabric test results.

Sample		1	2	3	4	5	6	7	8	9	10
A-A	F_(max)_ [N]	130.68	266.35	306.35	338.35	406.35	418.35	418.35	476.35	440.35	330.35
	ΔF_(max)_ [m]	0.0134	0.0205	0.0233	0.0242	0.0271	0.0304	0.0317	0.0342	0.0268	0.0358
B-B	F_(max)_ [N]	545.70	452.35	436.35	444.35	488.35	452.35	502.35	462.35	346.35	382.35
	ΔF_(max)_ [m]	0.0321	0.0248	0.0313	0.0264	0.0308	0.0294	0.0333	0.0318	0.0271	0.0255
A-B	F_(max)_ [N]	456.35	486.35	446.35	452.35	426.35	400.35	424.35	456.35	386.35	340.35
	ΔF(_max)_ [m]	0.0312	0.0348	0.0339	0.0274	0.0301	0.0318	0.0272	0.0321	0.0279	0.0251

**Table 5:** Soft 10 fabric test results.

Sample		1	2	3	4	5	6	7	8	9	10
A-A	F_(max)_ [N]	266.35	246.68	306.35	238.68	314.35	262.35	288.35	252.35	280.35	302.35
	ΔF(_max)_ [m]	0.0077	0.0287	0.0373	0.0289	0.0346	0.0330	0.0341	0.0322	0.0294	0.0335
B-B	F_(max)_ [N]	256.35	223.68	250.68	232.68	180.68	256.35	241.68	266.35	227.68	294.35
	ΔF(_max)_ [m]	0.0241	0.0289	0.0278	0.0299	0.0254	0.0316	0.0289	0.0292	0.0220	0.0311
A-B	F_(max)_ [N]	270.35	237.68	296.35	274.35	292.35	284.35	246.68	225.68	296.35	302.35
	ΔF(_max)_ [m]	0.0294	0.0263	0.0344	0.0337	0.0316	0.0309	0.0295	0.0296	0.0326	0.0317

## References

[1] Yacout D.M.M., Hassouna M.S. (2016). Identifying potential environmental impacts of waste handling strategies in textile industry. Environ. Monit. Assess.

[2] Saville B. (2002). Physical Testing of Textiles.

[3] Hu J. (2004). Structure and Mechanics of Woven Fabrics.

[4] Afroz F., Islam M.D.D. (2021). Study on mechanical property of woven fabrics made from 50/50 cotton-tencel blended siro yarn. Heliyon.

[5] Kireçci A., Kaynak H.K., Ince M.E. (2011). Comparative study of the quality parameters of knitted fabrics produced from siro spun single and two-ply yarns, Fibres Text. East. Eur..

[6] Mukhopadhyay S., Ghosh S. (2006). Bhaumik, Tearing and tensile strength behaviour of military khaki fabrics from greige to finished process. Int. J. Cloth. Sci. Technol..

[7] Cheng K.P.S., Sun M.N. (1993). Structure and properties of cotton siro spun yarn. Textil. Res. J..

[8] Nazir M.U., Shaker K., Nawab Y., Fazal M.Z., Khan M.I., Umair M. (2017). Investigating the effect of material and weave design on comfort properties of bilayer-woven fabrics. J. Textil. Inst..

[9] Nergis B.U., Candan C. (2006). Performance of Bouclé Yarns in Various Knitted Fabric Structures. Text. Res. J..

[10] Ramasamy K.A., Nalankilli G., Shanmugasundaram O.L. (2014). Properties of cotton, tencel and cotton/tencel blended ring- spun yarns. Indian J. Fibre Text. Res..

[11] Hossain M.d.H., Islam M., Chandra Dey S., Hasan N. (2021). An approach to improve the pilling resistance properties of three thread polyester cotton blended fleece fabric. Heliyon.

[12] Witczak E., Jasińska I., Lao M., Krawczyńska I., Kamińska I. (2021). The influence of structural parameters of acoustic panels textile fronts on their sound absorption properties. Appl. Acoust..

[13] You S., Cheng S., Yan H. (2009). The impact of textile industry on China’s environment. Int. J. Fash. Des. Technol. Educ..

[14] Wang M., Liu J., Chan H.-L., Choi T.-S., Yue X. (2016). Effects of carbon tariffs trading policy on duopoly market entry decisions and price competition: Insights from textile firms of developing countries. Int. J. Prod. Econ..

[15] Ranasinghe L., Jayasooriya V.M. (2021). Ecolabelling in textile industry: A review. Resour. Environ. Sustain..

[16] van der Velden N.M., Patel M.K., Vogtländer J.G. (2014). LCA benchmarking study on textiles made of cotton, polyester, nylon, acryl, or elastane. Int. J. Life Cycle Assess.

[17] Jamshaid H., Hussain U., Mishra R., Tichy M., Muller M. (2021). Turning textile waste into valuable yarn. Clean. Eng. Technol..

[18] Choudhary A.K., Goel A. (2013). Effect of Some Fabric and Sewing Conditions on Apparel Seam Characteristics. J. Text..

[19] Bhavesh R., Madhuri K., Sujit G., Sudhir M., Raichurkar P. (2018). Effect of Sewing Parameters on Seam Strength and Seam Efficiency. Trends Text. Eng. Fash. Technol..

[20] Abdel Megeid Z.M., Al-bakry M., Ezzat M. (2011). The influence of stitch length of weft knitted fabrics on the sewability. J. Am. Sci..

[21] Yesmin S., Hasan M., Miah S., Momotaz F., Idris M.A., Rashedul H. (2014). Effect of Stitch Length and Fabric Constructions on Dimensional and Mechanical Properties of Knitted Fabrics. World Appl. Sci. J..

[22] Smardzewski J. (1993). Sztywność dwustożkowych sprężyn tapicerskich. Przemysł Drzewny.

[23] Dzięgielewski S., Smardzewski J. (1995). Badania Formatek Sprężynowych Mebli Tapicerowanych.

[24] Linder-Ganz E., Yarnitzky G., Portnoy S., Yizhar Z., Gefen A. Real-time finite element monitoring of internal stresses in the buttock during wheelchair sitting to prevent sores: Verification and phantom results. Proceedings of the II International Conference on Computational Bioengineering.

[25] Schrodt M., Benderoth G., Kuhhorn A., Silber G. (2005). Hyperelastic description of polymer soft foams at finite deformations. Tech. Mech..

[26] Grujicic M., Pandurangan B., Arakere G., Bell W.C., He T., Xie X. (2009). Seat-cushion and soft-tissue material modeling and a finite element investigation of the seating comfort for passenger-vehicle occupants. Mater. Des..

[27] Smardzewski J., Wiaderek K. (2009). Non-linear stiffness characteristics of hyperelastic polyurethane foams, Annals of Warsaw Agricultural University-SGGW. For. Wood Technol..

[28] Lusiak A., Smardzewski J. (2010). Creative thinking in designing furniture for pre-school children. Annals of Warsaw University of Life Sciences SGGW. For. Wood Technol..

[29] Silber G., Alizadeh M., Salimi M. (2010). Large deformation analysis for soft foams based on hyperelasticity. J. Mech..

[30] Smardzewski J., Barańska-Woźny J., Wiaderek K., Prekrat S., Grbac I. Mechanical and biomechanical criteria in furniture designing for 60+ users. Proceedings of the International Conference.

[31] Wiaderek K., Smardzewski J. (2010). Numerical evaluation of seat hardness. Annals of Warsaw University of Life Sciences SGGW. For. Wood Technol..

[32] Wiaderek K., Smardzewski J. Modeling of foam seats in terms of comfortable relaxation furniture design. Proceedings of the International Conference.

[33] Wiaderek K., Matwiej Ł. (2016). Dettlaff M. Impact of structures of selected lounge furniture seats on the comfort of use, Annals of Warsaw University of Life Sciences SGGW. For. Wood Technol..

[34] Wiaderek K., Matwiej Ł., Jankowski K. (2015). Test for the auxetization of chosen polyurethane foams used in the production of seat bases for upholstered furniture. Annals of Warsaw University of Life Sciences-SGGW. For. Wood Technol..

[35] Smardzewski J., Matwiej Ł. (2013). Effects of Aging of Polyurethane Foams in the Context of Furniture Design. Drv. Ind..

[36] Babrauskas V., Krasny J.F. (1985). Fire Behavior of Upholstered Furniture.

[37] Hirschler M.M., Shakir S. (1991). Comparison of the Fire Performance of Va-rious Upholstered Furniture Composite Combinations (Fabric/Foam) in Two Rate of Heat Release Calorimeters: Cone and Ohio State University Instru-ments. J. Fire Sci..

[38] Lounis M., Leconte S., Rousselle C., Belzunces L.P., Desauziers V., Lopez-Cuesta J.-M., Julien J.M., Guenot D., Bourgeois D. (2019). Fireproofing of domestic upholstered furniture: Migration of flame retardants and potential risks. J. Hazard. Mater..

[39] Yang J., Rein G., Chen H., Zammarano M. (2020). Smoldering propensity in upholstered furniture: Effects of mock-up configuration and foam thickness. Appl. Therm. Eng..

[40] Chivas C., Guillaume E., Sainrat A., Barbosa V. (2009). Assessment of risks and benefits in the use of flame retardants in upholstered furniture in continental Europe. Fire Saf. J..

[41] Witkowska B., Witczak E. (2003). Odporność na ścieranie tkanin meblowych z udziałem przędz szenilowych w zależności od parametrów technologicznych przędzy szenilowej i tkaniny. Przegląd Włókienniczy + Tech. Włókienniczy.

[42] Zajkiewicz H. (1975). Budowa Tkanin.

[43] Lewiński J., Suszek H., Zawadzki J. (1975). Tkactwo. Część I.

[44] (1993). Textiles—Tensile Properties of Fabric.

[45] (1979). Materiały Tapicerskie dla Motoryzacji. Metody Badań. Oznaczenie Wytrzymałości Szwu na Rozciąganie.

[46] Kowalewski Z. (2000). Ćwiczenia Laboratoryjne z Wytrzymałości Materiałów.

